# Critical Requirement of Senescence-Associated CCN3 Expression in CD44-Positive Stem Cells for Osteoarthritis Progression

**DOI:** 10.3390/ijms26199630

**Published:** 2025-10-02

**Authors:** Janvier Habumugisha, Ryuichiro Okuda, Kazuki Hirose, Miho Kuwahara, Ziyi Wang, Mitsuaki Ono, Hiroshi Kamioka, Satoshi Kubota, Takako Hattori

**Affiliations:** 1 Department of Biochemistry and Molecular Dentistry, Okayama University Graduate School of Medicine, Dentistry and Pharmaceutical Sciences, 5-1 Shikata-cho, 2-chome, Kita-ku, Okayama 700-8525, Japan; januhabzan@gmail.com (J.H.); ryuichiro.okuda@gmail.com (R.O.); hirose6342@gmail.com (K.H.); kuwahara.miho@s.okayama-u.ac.jp (M.K.); kubota1@md.okayama-u.ac.jp (S.K.); 2 Department of Orthodontics, Okayama University Graduate School of Medicine, Dentistry and Pharmaceutical Sciences, Okayama 700-8525, Japan; kamioka@md.okayama-u.ac.jp; 3 Department of Orthopedic Surgery, Okayama University Graduate School of Medicine, Dentistry and Pharmaceutical Sciences, Okayama 700-8558, Japan; 4 Department of Occlusal and Oral Functional Rehabilitation, Okayama University Graduate School of Medicine, Dentistry and Pharmaceutical Sciences, Okayama 700-8525, Japan; 5 Department of Molecular Biology and Biochemistry, Okayama University Graduate School of Medicine, Dentistry and Pharmaceutical Sciences, Okayama 700-8558, Japan; wangziyi@s.okayama-u.ac.jp; 6 Department of Oral Rehabilitation and Regenerative Medicine, Okayama University Graduate School of Medicine, Dentistry and Pharmaceutical Sciences, Okayama 700-8525, Japan; mitsuaki@md.okayama-u.ac.jp

**Keywords:** articular, cartilage, mesenchymal stem cells, nephroblastoma overexpressed protein, osteoarthritis

## Abstract

Osteoarthritis (OA) is a degenerative joint disease characterized by progressive cartilage breakdown, synovial inflammation, and subchondral bone remodeling. Previous studies have shown that cellular communication network factor 3 (CCN3) expression increases with age in cartilage, and its overexpression promotes OA-like changes by inducing senescence-associated secretory phenotypes. This study aimed to investigate the effect of *Ccn3* knockout (KO) on OA development using a murine OA model. Destabilization of the medial meniscus (DMM) surgery was performed in wild-type (WT) and *Ccn3*-KO mice. Histological scoring and staining were used to assess cartilage degeneration and proteoglycan loss. Gene and protein expressions of catabolic enzyme (*Mmp9*), hypertrophic chondrocyte marker (*Col10a1*), senescence marker, and cyclin-dependent kinase inhibitor 1A (*Cdkn1a*) were evaluated. Single-cell RNA sequencing (scRNA-seq) data from WT and *Sox9*-deficient cartilage were reanalyzed to identify *Ccn3*^+^ progenitor populations. Immunofluorescence staining assessed CD44 and Ki67 expression in articular cartilage. The effects of *Ccn3* knockdown on IL-1β-induced *Mmp13* and *Adamts5* expression in chondrocytes were examined *in vitro*. *Ccn3* KO mice exhibited reduced cartilage degradation and catabolic gene expression compared with WT mice post-DMM. scRNA-seq revealed enriched *Ccn3*-*Cd44* double-positive cells in osteoblast progenitor, synovial mesenchymal stem cell, and mesenchymal stem cell clusters. Immunofluorescence showed increased CCN3^+^/CD44^+^ cells in femoral and tibial cartilage and meniscus. Ki67^+^ cells were significantly increased in DMM-treated *Ccn3* KO cartilage, mostly CD44^+^. In vitro *Ccn3* knockdown attenuated IL-1β-induced *Mmp13* and *Adamts5* expressions in chondrocytes. *Ccn3* contributes to OA pathogenesis by promoting matrix degradation, inducing hypertrophic changes, and restricting progenitor cell proliferation, highlighting *Ccn3* as a potential therapeutic target for OA.

## 1. Introduction

Osteoarthritis (OA) is one of the most prevalent degenerative joint disorders, affecting over 25% of adults [1], and involves a gradual breakdown of articular cartilage, increased density and thickening of the subchondral bone, formation of osteophytes, and varying levels of inflammation in the synovial membrane. Other pathological features may include damage to the knee ligaments and menisci, as well as enlarged joint capsules [1,2]. Despite its widespread prevalence, the molecular mechanisms driving OA progression remain incompletely understood, and effective disease-modifying therapies are still lacking. Recently, innovative disease-modifying strategies, such as the use of biomaterials, regenerative scaffolds, and cell-based therapies, have shown promise in preclinical and early clinical studies, but their efficacy remains under investigation. Given these limitations, it is crucial to gain a better understanding of the biological mechanisms underlying OA [3,4,5]. OA is a multifactorial disease influenced by genetic, aging, environmental, and mechanical factors. These factors disrupt joint homeostasis, leading to changes in cartilage, bone, and surrounding tissues [1,6,7].

Cellular communication network factor 3 (CCN3), a matricellular protein, regulates diverse biological processes, including cell proliferation, angiogenesis, differentiation, and extracellular matrix (ECM) remodeling. CCN3 plays a critical role in maintaining tissue homeostasis and modulating the cellular environment in bone and cartilage [8,9,10,11,12,13,14,15]. It has recently been proposed as a potential modulatory factor in joint diseases [8,9]. CCN3 has been implicated in several key aspects of OA, particularly in its effects on chondrocytes, the primary cartilage cells. Some studies suggest that CCN3 may contribute to cartilage degradation by modulating chondrocyte activity, enhancing inflammatory signaling pathways, and promoting ECM breakdown. Notably, *Ccn3* expression increases with age, and its overexpression induces cellular senescence and cell cycle arrest. In vivo models further demonstrate that *Ccn3* overexpression leads to OA-like changes in cartilage, accompanied by increased accumulation of senescent cells [8,16]. Additionally, CCN3 has been implicated as a key player in the pathogenesis of rheumatoid arthritis [9,17,18]. On the other hand, other research indicates that CCN3 may have a protective role in cartilage by modulating repair mechanisms and limiting inflammatory damage, depending on the cellular context and local environment [19,20]. This dual potential of CCN3 in OA makes it a controversial yet promising target for further investigation, as understanding its precise function could provide valuable insights into developing more effective therapeutic strategies for OA management.

One critical factor in OA progression is CD44, a transmembrane glycoprotein that serves as the primary receptor for hyaluronan. CD44 plays a key role in chondrocyte metabolism, ECM homeostasis, and inflammation. Its interactions with hyaluronan influence cellular signaling, which is crucial for cartilage integrity [21,22,23]. Recent work on stem progenitors/stem cells in articular and growth plate cartilage identified Cd44 as a mesenchymal stem cell (MSC) marker [24,25,26,27,28]. Additionally, single-cell RNA sequencing (scRNA-seq) analyses have identified several types of progenitors or stem cells in articular and growth plate cartilage, as well as in bone marrow [29,30,31,32,33,34].

Cell proliferation and subsequent repair are essential for cartilage maintenance, with Ki67 serving as a key marker of chondrocyte proliferation and regenerative potential. In OA chondrocytes, reduced Ki67 expression is observed and may reflect impaired chondrocyte renewal and compromised tissue repair [35]. This diminished proliferative capacity is often associated with increased chondrocyte senescence, which contributes to accelerated cartilage degradation [36,37]. Notably, *Ccn3* has been implicated in promoting chondrocyte senescence, possibly through inflammatory signaling and ECM remodeling, thereby exacerbating OA progression [16].

In this study, the role of *Ccn3* in OA was investigated using a mouse model of joint degeneration. Specifically, the effects of *Ccn3* deletion on cartilage degradation, chondrocyte proliferation, and CD44 expression were examined through histological, molecular, and scRNA-seq analyses. The findings provide new insights into the molecular processes that contribute to OA progression and highlight CCN3 as a potential therapeutic target.

## 2. Results

### 2.1. Ccn3 WT (DMM) Exhibits Severe Cartilage Degradation, While Ccn3 KO (DMM) Demonstrates Protective Effects

**Figure 1:**
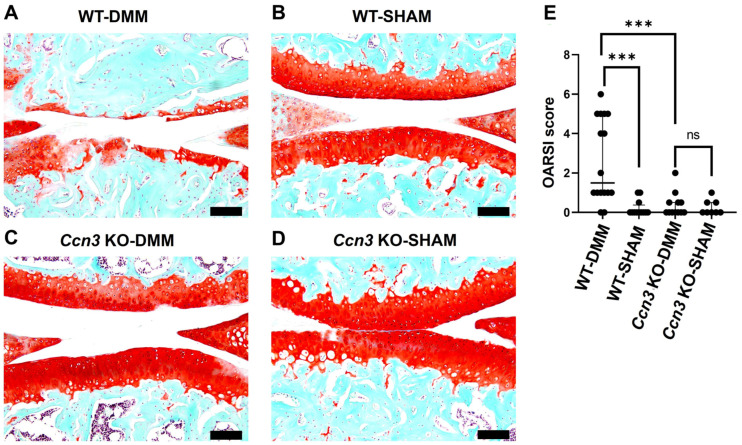
*Ccn3* deletion attenuates OA-associated cartilage degradation. (**A**–**D**) Representative Safranin O/Fast Green-stained sagittal sections of knee joints from *Ccn3* WT and KO mice subjected to DMM or sham surgery. WT-DMM joints exhibited marked loss of proteoglycans and cartilage structural integrity, whereas *Ccn3* KO-DMM joints retained higher Safranin O staining and preserved cartilage morphology, comparable to sham controls. Scale bar: 100 μm. (**E**): Quantification of cartilage damage using the Osteoarthritis Research Society International (OARSI) scoring system confirmed significantly increased scores in WT-DMM mice compared to *Ccn3* KO-DMM and sham groups (*** *p* < 0.001; ns, not significant). Data are presented as individual values with the median and interquartile range (IQR). Statistical analysis was performed using the Kruskal–Wallis test, followed by Dunn’s multiple comparisons. WT-DMM (sample number “*n*” = 4), WT-Sham (*n* = 3), *Ccn3* KO-DMM (*n* = 3), and *Ccn3* KO-Sham (*n* = 2).

DMM surgery was performed on the articular cartilage of *Ccn3* wild-type (WT) and *Ccn3* knockout (KO) mice to elucidate the pathological link between *Ccn3* expression and OA progression. Eight weeks post-surgery, knee joint tissues were harvested for analysis. Safranin O staining revealed significant proteoglycan loss and structural degradation in the *Ccn3* WT (DMM) group (Figure 1A) compared to the *Ccn3* WT (sham) group (Figure 1B), indicating advanced cartilage damage following DMM. Notably, *Ccn3* KO (DMM) mice (Figure 1C) exhibited less cartilage degradation than their WT counterparts, suggesting that *Ccn3* deletion protects against OA-induced cartilage breakdown (Figure 1A–D). Consistently, OARSI scoring demonstrated significantly higher scores in the WT (DMM) group than the KO (DMM) and sham-operated groups (Figure 1E).

Together, these results demonstrate that *Ccn3* depletion alleviates murine OA progression by reducing degenerative changes in the articular cartilage.

### 2.2. Ccn3 Deletion Attenuates the Expression of Cartilage-Degrading Enzymes in DMM-OA

**Figure 2:**
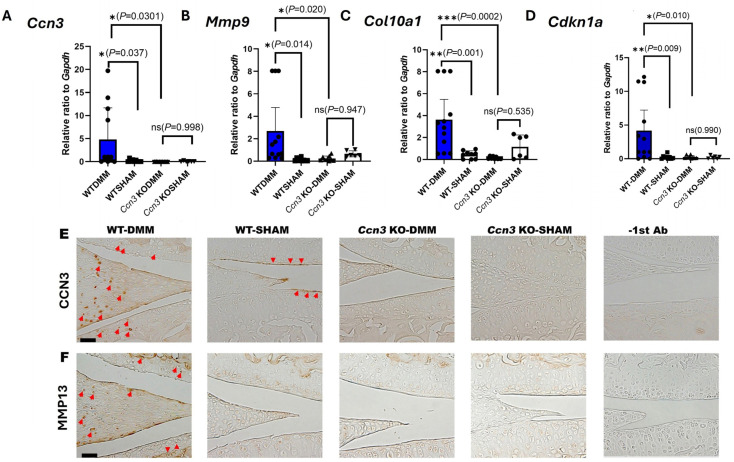
*Ccn3* deletion attenuates the expression of cartilage-degrading enzymes in OA (RT-qPCR and immunohistochemistry). (**A**–**D**) RT-qPCR analysis of cartilage from *Ccn3*-WT and *Ccn3*-KO mice subjected to DMM-induced OA or sham surgery. Gene expression levels of *Ccn3*, *Mmp9*, *Col10a1*, and *Cdkn1a*/p21 were normalized to *Gapdh*. WT-DMM mice exhibited significantly higher expression of these catabolic and hypertrophic markers, as well as *Cdkn1a*/p21, compared to sham controls, indicating active cartilage degradation. * *p* < 0.05, ** *p* < 0.01, *** *p* < 0.001, ns = not significant. (**E**,**F**) Immunohistochemical analysis showed increased staining of CCN3 (arrow) and MMP-13 (arrowhead) in *Ccn3* WT-DMM compared to WT-Sham, indicating elevated cartilage degradation. In contrast, *Ccn3* KO-DMM exhibited minimal staining for MMP-13, comparable to *Ccn3* KO-Sham, suggesting reduced matrix breakdown. CCN3 staining was absent in both *Ccn3* KO groups, confirming successful knockout. Scale bar = 50 µm.

Then, gene expressions in articular cartilage from *Ccn3* WT-DMM and sham groups, as well as *Ccn3* KO-DMM and sham groups, were assessed by RT-qPCR, revealing distinct differences among the groups. In *Ccn3* WT-DMM cartilage, the expression levels of *Ccn3* (Figure 2A), *Mmp9* (Figure 2B), *Col10a1* (Figure 2C), and *Cdkn1a* (Figure 2D) were significantly upregulated compared to *Ccn3* WT-Sham, consistent with previous findings [8,27]. *Ccn3* KO-DMM cartilage showed no significant changes in *Mmp9* (Figure 2B), *Col10a1* (Figure 2C), or *Cdkn1a* (Figure 2D) expression in contrast to *Ccn3* KO-Sham, suggesting that cartilage is protected from DMM-induced degradation in the absence of *Ccn3*. Further comparison between *Ccn3* WT (DMM) and *Ccn3* KO (DMM) revealed significantly lower *Mmp9* (Figure 2B), *Col10a1* (Figure 2C), and *Cdkn1a*, encoded p21 (Figure 2D and Tables S1–S5) expressions in *Ccn3* KO (DMM), suggesting that *Ccn3* deletion may attenuate matrix degradation and reduce senescence-associated responses in cartilage.

Immunohistochemistry was performed to evaluate cell localization and the accumulation of key proteins within the cartilage tissue. Strong CCN3 staining was detected in both femoral and tibial articular cartilage, as well as in the meniscus, of *Ccn3* WT-DMM joints, confirming active CCN3 expression in OA cartilage. In contrast, CCN3 accumulation was observed in the superficial layer of the articular cartilage in *Ccn3* WT-Sham. No staining was observed in the *Ccn3* KO-DMM group, confirming successful *Ccn3* gene knockout (Figure 2E). In *Ccn3* WT-DMM joints, increased MMP-13 staining (Figure 2F) was observed compared to *Ccn3* WT-Sham, indicating enhanced cartilage degradation associated with DMM. These differences were not detected in *Ccn3* KO-DMM joints relative to their sham counterparts, suggesting reduced cartilage breakdown in the absence of *Ccn3*. Furthermore, when comparing *Ccn3* WT-DMM to *Ccn3* KO-DMM, significantly lower levels of MMP-13 staining (Figure 2F) were observed in the KO group, reinforcing the protective effect of *Ccn3* deletion.

### 2.3. Single-Cell RNA Sequencing Reveals That Ccn3-Expressing Clusters Co-Express Cd44 in Postnatal Day 13 Mouse Cartilage

**Figure 3:**
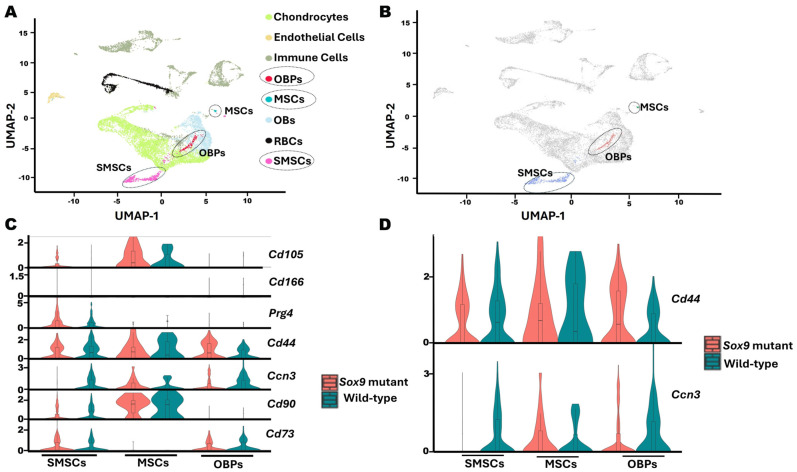
scRNA-seq of postnatal day 13 mouse cartilage indicates Ccn3-expressing clusters also express *Cd44*. (**A**) UMAP visualization shows 8 identified cell clusters from combined *Sox9* mutant and wild-type (WT) cartilage samples. (**B**) UMAP plot displaying the CCN3-expressing clusters (colors represent clusters). SMSCs, MSCs, and OBPs are the major clusters exhibiting enriched *Ccn3* expressions. (**C**) Violin plots illustrate the expression of *Ccn3* and key chondroprogenitor-associated markers (*Cd44*, *Cd90* (*Thy1*), *Cd73* (*Nt5e*), *Cd105* (*Eng*), *Cd166* (*Alcam*), and *Prg4*) across *Ccn3*-enriched clusters. Notably, *Cd44* was the only marker consistently expressed in all *Ccn3*-positive clusters. (**D**) Violin plots of *Ccn3* and *Cd44* expression specifically within the *Ccn3*-expressing clusters (SMSCs, MSCs, and OBPs) compared with *Sox9* mutant (red) and WT (blue) samples. These analyses demonstrate the rationale for cluster selection and marker prioritization in evaluating the relationship between *Ccn3* and progenitor identity.

To identify the *Ccn3*-expressing cell population in DMM-induced articular cartilage, a publicly available single-cell RNA-seq dataset from a *Sox9*-mutated OA-like model and WT controls was re-analyzed. This analysis revealed enriched *Ccn3* expression within three primary clusters: Osteoblast Progenitors (OBPs), Synovial Mesenchymal Stem Cells (SMSCs), and Mesenchymal Stem Cells (MSCs) (Figure 3A,B). The expressions of established progenitor markers associated with articular cartilage and skeletal development, including *Cd44*, *Cd90* (*Thy1*), *Cd73* (*Nt5e*), *Cd105* (*Eng*), *Cd166* (*Alcam*), and *Prg4,* were examined to investigate the potential chondrocyte progenitor identity of these *Ccn3*-expressing clusters [24,25,26,38,39]. Among these markers, only *Cd44* was consistently expressed across all *Ccn3*-positive clusters, suggesting a potential regulatory relationship between *Ccn3* and *Cd44* (Figure 3C,D).

The *Sox9*-mutant OA model and WT dataset were then assessed to explore the relationship between *Ccn3* and *Cd44* expressions within the identified clusters. Among *Ccn3*-expressing clusters, MSCs showed high *Ccn3* expression in *Sox9*-mutant OA. In contrast, *Ccn3* expression was lower in the SMSC and OBP clusters in *Sox9*-mutant OA than WT (Figure 3D), suggesting that CCN3^+^ MSCs, but not SMSCs and OBPs, increase their response to OA-like changes due to *Sox9* mutation.

### 2.4. Colocalization of CCN3 and CD44 in Ccn3 WT-DMM Samples

**Figure 4:**
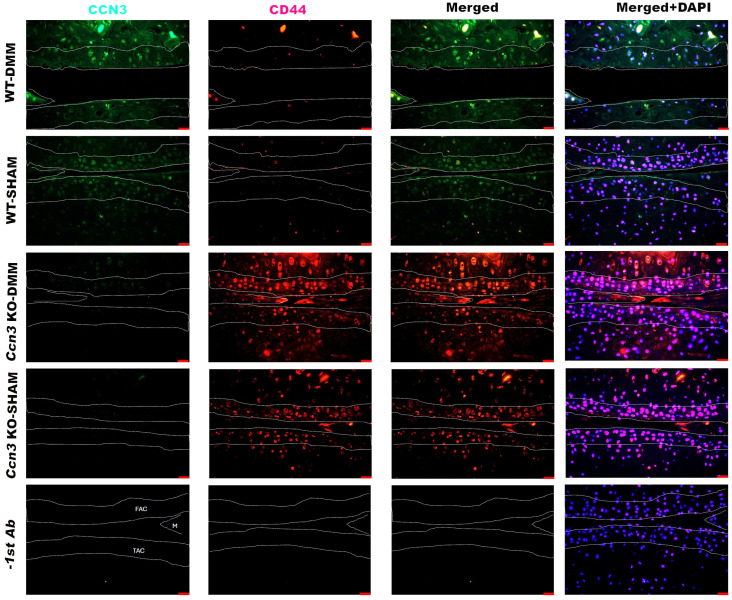
Colocalization of CCN3^+^ and CD44^+^ cells in *Ccn3* WT-DMM samples revealed by immunofluorescence. Immunofluorescence staining shows the expression patterns of CCN3 (green) and CD44 (red), with merged images (right panels) indicating colocalization in the articular cartilage and subchondral bone regions. Colocalization is most prominent in *Ccn3* WT-DMM samples, suggesting a potential interaction between CCN3 and CD44 in osteoarthritis pathogenesis. Dotted lines indicate the boundaries of the cartilage and subchondral bone. FAC: femoral articular cartilage; TAC: tibial articular cartilage; M: meniscus. Experimental groups: *Ccn3* KO-DMM (*n* = 3), WT-DMM (*n* = 4), *Ccn3* KO-Sham (*n* = 2), and WT-Sham (*n* = 3). Scale bar: 20 µm.

Double immunofluorescence staining for CCN3 and CD44 was performed to clarify whether the amplified cells exhibit the characteristics of stem/progenitor cells of articular chondrocytes, which express CD44 as a marker protein [26,39,40,41,42]. The immunofluorescent images demonstrated that most CCN3-expressing cells in both femoral and tibial cartilage, as well as subchondral bone of *Ccn3* WT-DMM, express CD44 (Figure S3). This suggests a potential function of CCN3 in the stem/progenitor cell of cartilage and skeletal tissues (Figure 4). In *Ccn3* WT-DMM, CD44^+^ stem/progenitor cells decreased compared to *Ccn3* WT-SHAM, *Ccn3* KO-DMM, and *Ccn3* KO-SHAM, indicating negative effects of CCN3 on CD44^+^ stem/progenitor cells upon DMM.

As noted, the surface of the articular superficial zone and meniscus showed CCN3 signals in WT-sham, and CCN3^+^ CD44^+^ cells were observed in these regions (Figure 4), indicating the physiological role of CCN3 in maintaining cartilage homeostasis.

### 2.5. Ccn3 KO-DMM Displays Higher Chondrocyte Proliferation than Ccn3 WT-DMM

**Figure 5:**
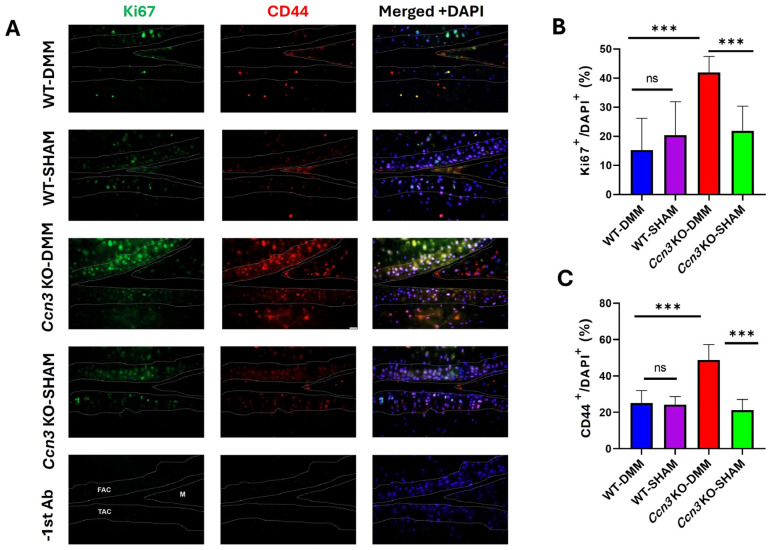
Double immunofluorescence staining of CD44 and Ki67 in WT and KO mice under OA and sham conditions. (**A**) Representative immunofluorescence images showing co-staining of CD44 (red) and Ki67 (green) in articular cartilage from *Ccn3* WT and *Ccn3* KO mice subjected to OA induction or sham surgery. Increased Ki67 staining is observed in the *Ccn3* KO-DMM group compared to both the *Ccn3* KO-Sham and *Ccn3* WT (DMM) groups, suggesting enhanced proliferative activity in the absence of *Ccn3* under OA conditions. The dotted line indicates FAC, TAC, and M (FAC, femoral articular cartilage; TAC, tibial articular cartilage; M, meniscus). (**B**,**C**) Percentages of Ki67^+^ and CD44^+^ cells (green/red) relative to the total number of nucleated cells (DAPI^+^, blue) within the FAC, TAC, and M. WT-DMM (*n* = 4), WT-Sham (*n* = 3), *Ccn3* KO-DMM (*n* = 3), and *Ccn3* KO-Sham (*n* = 2). *p*  <  0.001 (***), ns = not significant, scale bar: 20 µm.

To assess whether CCN3 affects the proliferation of CD44^+^ stem/progenitor cells, Ki67 expression was evaluated as a marker of cell proliferation via immunofluorescence. A higher number of Ki67^+^-CD44^+^ cells was observed in the articular cartilage of *Ccn3* KO-DMM mice compared with *Ccn3* WT-DMM mice, suggesting that *Ccn3* induction via DMM may inhibit stem/progenitor cell proliferation and impair cartilage repair. Additionally, Ki67 expression was higher in *Ccn3* KO-DMM than in *Ccn3* KO-Sham, suggesting that DMM stimulation in articular cartilage may promote the proliferation of Cd44^+^ stem/progenitor cells. This also highlights a potential role of CCN3 in regulating the cell cycle of stem/progenitor cells, not only in OA but also in maintaining homeostasis in healthy cartilage (Figure 5A–C).

### 2.6. Ccn3 Knockdown Attenuates IL-1β-Induced Expression of Cartilage Degradation and Senescence Markers in RCS Cells

**Figure 6:**
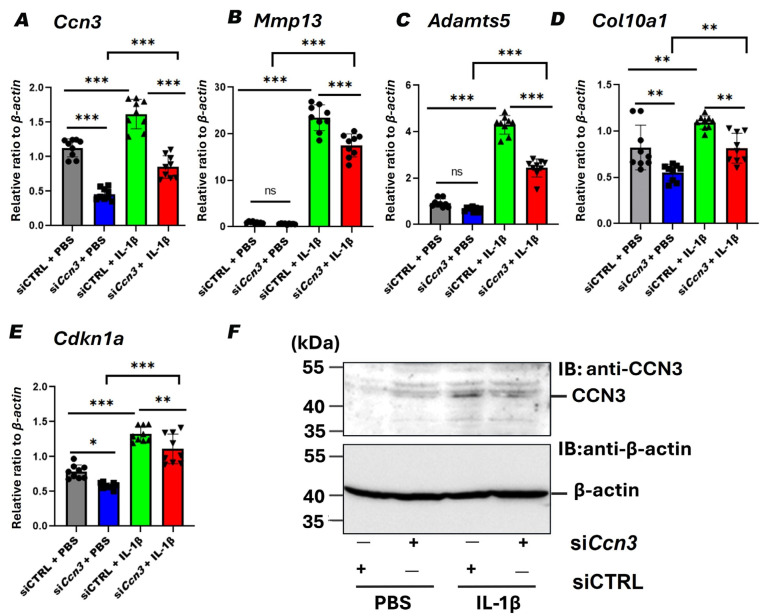
*Ccn3* knockdown attenuates IL-1β-induced expression of cartilage degradation and senescence markers in RCS cells in vitro. (**A**–**F**) RT-qPCR and Western blot analyses of RCS chondrocyte-like cells transfected with either control siRNA (siCTRL) or Ccn3-targeting siRNA (si*Ccn3*) at 100 nM. After 48 h, cells were stimulated with IL-1β (5 ng/mL) for an additional 24 h to mimic osteoarthritic conditions. (**A**–**E**) Messenger RNA expression of *Ccn3*, *Mmp13*, *Adamts5*, *Col10a1*, and *Cdkn1a*, respectively. IL-1β significantly upregulated all five genes, and these increases were attenuated by si*Ccn3*. All expression values were normalized to *β-actin*. (**F**) Representative Western blot showing CCN3 protein (40–55 kDa) with β-actin as a loading control. IL-1β increased CCN3 protein levels, which were effectively reduced by siCcn3. Data are presented as mean ± SD. Statistical analysis was performed using one-way ANOVA, followed by Dunnett’s post hoc test. * *p* < 0.05; ** *p* < 0.01; *** *p* < 0.001; ns, not significant. (*n* = 3 per group; all experiments performed in triplicate).

To clarify whether *Ccn3* downregulation via siRNA attenuates the expression of chondrocyte-degrading enzymes induced by IL-1β *in vitro*, rat chondrosarcoma (RCS) cells were treated with IL-1β with or without *Ccn3* knockdown, and gene expression was analyzed. IL-1β stimulation significantly increased the expressions of *Ccn3*, *Mmp13*, *Adamts5*, *Col10a1*, and *Cdkn1a* that encode the senescence marker p21 (Figure 6A–E), mimicking OA-like catabolic, hypertrophic, and senescent phenotypes. *Ccn3* knockdown markedly attenuated those IL-1β-induced gene expressions, indicating that *Ccn3* is required for activating catabolic, hypertrophic, and senescence pathways under inflammatory conditions (Figure 6A–E). *Ccn3* induction and knockdown by siRNA were also confirmed with Western blot analysis (Figure 6F). These findings collectively support a key role for *Ccn3* in promoting cartilage degeneration and cellular senescence in OA.

## 3. Discussion

Unraveling the molecular mechanisms underlying OA pathogenesis is essential for developing targeted strategies to slow or prevent disease progression [43,44,45]. This study identifies *Ccn3* as a key regulator of chondrocyte proliferation, matrix interaction, and metabolism, as supported by in vivo, in vitro, and single-cell transcriptomic evidence.

This study demonstrates that the presence of *Ccn3* contributes to OA-induced cartilage degeneration, whilst its absence confers significant cartilage protection. Previous studies have highlighted the dual and context-dependent roles of *Ccn3* in cartilage biology [8,9,12,16,17,19]. In fact, our previous study showed that *Ccn3* overexpression promotes a senescence-associated secretory phenotype (SASP) and extracellular matrix (ECM) degradation in aging cartilage [16]. Additionally, elevated CCN3 expression in articular cartilage was found to be associated with OA in human hip joints. CCN3 levels in cartilage were significantly and positively correlated with the Mankin score, a well-established histological marker of OA severity [8]. Conversely, some studies have reported that *Ccn3* may play a protective role in articular cartilage [12,19]; for instance, Roddy et al. [12] reported that the global disruption of *Ccn3* results in spontaneous OA-like changes in aged male mice. Notably, our study differs in the use of the DMM model to simulate post-traumatic OA, allowing for a direct comparison between *Ccn3* KO and WT mice. This experimental design assesses whether the absence of *Ccn3* confers protection in a surgically induced OA model, which is free from age-related systemic effects. Collectively, these findings suggest that the role of *Ccn3* in OA may be highly context-dependent, influenced by factors such as disease models and age-specific events.

Mechanistically, in vivo data demonstrated that *Ccn3* WT cartilage subjected to DMM surgery exhibited significantly increased cartilage degradation compared to both the sham and *Ccn3* KO-DMM groups, as evidenced by Safranin O staining and elevated expressions of *Mmp9* and *Col10a1*, key markers of matrix breakdown and chondrocyte hypertrophy. In contrast, these markers showed no significant difference between *Ccn3* KO-DMM and *Ccn3* KO sham groups, indicating reduced catabolic and hypertrophic responses in the absence of *Ccn3*. Similarly, *in vitro* knockdown of *Ccn3* in RCS cells decreased the expression of these catabolic genes compared to the control, further proving the protective effect of *Ccn3* suppression. Immunohistochemistry revealed consistently strong MMP-13 protein expression in the cartilage and meniscus of WT OA mice, which was markedly attenuated in KO mice, confirming the role of CCN3 in extracellular matrix degradation. Previous studies reported elevated *Ccn3* expression in joint diseases, including rheumatoid arthritis (RA) and OA [8,9,17], which was associated with inflammation and upregulation of matrix-degrading enzymes. In agreement with these studies, our findings suggest that CCN3 promotes MMP-13-mediated matrix breakdown in osteoarthritic joints. These findings suggest restraining *Ccn3* as a potential therapeutic target in OA.

Interestingly, *Ccn3* KO (DMM) cartilage exhibited significantly higher percentages of Ki67^+^ and CD44^+^ cells than all other groups, indicating enhanced chondrocyte proliferation and matrix interaction in the absence of *Ccn3* under OA conditions. Conversely, WT cartilage showed a marked reduction in these markers following DMM surgery, suggesting that *Ccn3* negatively regulates these processes in the context of joint injury. Furthermore, immunofluorescence analysis revealed that Ccn3-expressing cells expressed CD44 in WT OA cartilage, suggesting that CCN3^+^ CD44^+^ chondrocytes may represent a non-proliferative population, potentially associated with the cellular senescence of SMSCs, MSCs, and OBPs, or with catabolic activity [9,16]. This interpretation is in line with findings in other cell types; for example, in clear cell renal cell carcinoma, *Ccn3* expression was inversely correlated with the proliferation marker Ki67, and *Ccn3*-expressing cells exhibited reduced proliferative capacity [15]. Similar antiproliferative effects of *Ccn3* have been reported in glioma and melanocyte models [46,47], highlighting its role as a context-dependent modulator of cell fate.

CD44 itself plays complex roles in cartilage biology, contributing to both normal tissue maintenance and OA progression via interactions with hyaluronan [48,49,50,51]. These interactions influence chondrocyte proliferation, inflammatory responses, and matrix remodeling. Therefore, CD44 and CCN3 colocalization in OA cartilage may reflect a pathogenic chondrocyte subpopulation characterized by diminished proliferative activity rather than a matrix-maintaining phenotype. This interpretation is further supported by our single-cell transcriptomic re-analysis of cartilage from P13 WT mice and *Sox9* mutant mice, a model commonly used to represent an OA-like phenotype [52,53,54]. *Cd44* was consistently co-expressed across all *Ccn3*-positive clusters, highlighting a complex, cell-type-specific regulatory relationship.

The interplay between *Ccn3* and senescence-associated pathways may underlie the phenotypes observed in our model. In our previous work, we demonstrated that *Ccn3* acts as a positive regulator of key senescence markers by upregulating both p53 and p21 (*Cdkn1a*) gene expression in chondrocytes, thereby promoting cellular senescence [16]. Given the central role of these genes in mediating cell cycle arrest and senescence in response to stress signals, their expression levels serve as critical indicators of cellular aging and inflammatory status [55,56,57]. In the current study, under IL-1β stimulation, *Ccn3* knockdown significantly reduced *Cdkn1a* expression, possibly indicating reduced senescence and a shift toward a more reparative phenotype. In vivo analysis consistently revealed that *Cdkn1a* expression was markedly upregulated in *Ccn3* WT-DMM cartilage, whereas *Ccn3* KO-DMM cartilage showed no significant change compared to sham controls, indicating that *Ccn3* deletion protects cartilage from DMM-induced senescence. Furthermore, *Cdkn1a* levels in *Ccn3* KO-DMM cartilage were significantly lower than in WT-DMM, highlighting the role of *Ccn3* in promoting senescence-associated responses in injured cartilage. Thus, targeting *Ccn3* could offer a therapeutic approach to modulate inflammation-induced senescence and restore tissue homeostasis. These findings suggest that *Ccn3* deletion mitigates inflammation-induced senescence [9] while enhancing cell proliferation, collectively promoting cartilage repair. Mechanistically, these effects are consistent with *Ccn3* acting through the p53–p21 pathway, where *Ccn3*-driven activation enforces a senescent, pro-inflammatory chondrocyte state, while its suppression promotes proliferation and tissue repair.

Previous studies have demonstrated that, in RA patients, *Ccn3* activates the senescence pathway in synoviocytes and promotes osteoclast differentiation [9], indicating that *Ccn3* may induce joint destruction via multiple mechanisms. Furthermore, elevated serum CCN3 levels in RA patients correlate with inflammatory cytokines, such as IL-6, which is critical in RA pathogenesis [17].

### Limitations

The limitations of this study include the following: the sample size was limited, and data from male and female mice were combined. Additionally, on postnatal day 13 (P13), *Sox9*-knockout cartilage was used to model OA-like phenotypes, which may differ from the adult or post-traumatic OA model employed in this study. Nevertheless, consistent trends observed across histological, molecular, and *in vitro* analyses support the robustness of our findings. Future investigations with larger, sex-balanced cohorts and additional adult OA models are warranted to validate these results and further elucidate the underlying mechanisms.

## 4. Materials and Methods

### 4.1. Animal Model and Experimental Groups

*Ccn3*-deficient mice were generated by replacing exons 1, 2, and a distal portion of exon 3 with a neomycin resistance cassette, which was obtained from Dr. Ryusuke Yoshida (Okayama University) [58,59]. The sequences of the PCR primers used for genotyping were 5′-TGA ATG AAC TGC AGG ACG AG-3′ and 5′-AAT ATC ACG GGT AGC CAA CG-3′, which detects the NeoR cassette, and 5′-GGC TTC CTG CTC TTC CAT CTC TTA-3′ and 5′-CCT TCT CTA GGC GGC AAG TGA CCT-3′, which detects the targeted region of *Ccn3* (Figure S1).

This study utilized four-month-old male and female C57BL/6J mice (both wild-type [WT] and *Ccn3* knockout [KO]). All animals were housed under standardized conditions (12-h light/dark cycle) with ad libitum access to food and water. All procedures were approved by the Institutional Animal Care and Use Committee of Okayama University (approval number: OKU-2023616 and OKU-2023608).

The mice were randomly assigned to four experimental groups: *Ccn3* knockout with destabilization of the medial meniscus (*Ccn3* KO (DMM); *n* = 3, 2 females and 1 male), wild-type with DMM (WT (DMM); *n* = 4, 3 females and 1 male), *Ccn3* knockout with sham surgery (*Ccn3* KO (Sham); *n* = 2, 1 female and 1 male), and wild-type with sham surgery (WT (Sham); *n* = 3, 2 females and 1 male).

To establish the DMM model of osteoarthritis, surgery was performed on both knee joints. Under general anesthesia, the medial meniscotibial ligament was transected bilaterally in WT and *Ccn3* KO mice to induce joint destabilization [60]. In the sham-operated groups, skin incisions and wound closure were performed on both knees without meniscal destabilization.

Due to a limited number of male mice, we combined data from male and female mice to increase the sample size and statistical power. This approach can be justified by findings in other tissues, where no sex-related differences were observed in phenotypes associated with *Ccn3* deficiency [61].

### 4.2. Histology and Safranin O Staining

Mice were euthanized using 100% CO_2_. Death was confirmed by the absence of movement, respiration, and cardiac activity. Knee joints were then collected. The tissues were initially fixed in 4% paraformaldehyde/PBS for 48 h, decalcified with Osteosoft (Merck, Darmstadt, Germany) until becoming sufficiently soft for sectioning, dehydrated, embedded in paraffin, and sectioned at 7 µm thickness. To assess cartilaginous matrix, Safranin-O/fast green staining was performed [8].

### 4.3. Immunofluorescence and Immunohistochemistry

Targeted protein labeling was performed using either fluorescent dye or diaminobenzidine (DAB). Tissue sections were baked at 60 °C for 1 h, followed by deparaffinization and rehydration. Sections were then incubated with hyaluronidase for 30 min to facilitate antigen exposure. Afterward, the slides were rinsed in Tris-buffered saline with 0.1% Tween-20 (TBST), blocked using Blocking One-P (Nacalai Tesque, Kyoto, Japan) for 1 h at room temperature, and then incubated overnight at 4 °C with primary antibodies against CCN3 (1:100; provided by Dr. Sasaki, Oita University) [8,16], anti-CD44 (1:100; Cell Signaling Technology, Danvers, MA, USA), and anti-Ki67 (1:100; Proteintech Group, Inc., Rosemont, IL, USA). Following TBST washes, sections were incubated with secondary antibodies: Alexa Fluor^®^ 568-conjugated goat anti-mouse IgG and Alexa Fluor^®^ 488-conjugated goat anti-rabbit IgG (Life Technologies, Carlsbad, CA, USA).

### 4.4. Single-Cell RNA Sequencing Data Processing and Clustering

A secondary analysis of published single-cell RNA-sequencing (scRNA-seq) data (NCBI GEO accession GSE162033) was performed to characterize *Ccn3* expression and its relationship with progenitor-cell markers in murine cartilage. Two post-natal day-13 (P13) samples were analyzed: wild-type epiphyses (GSM4930081, WT-P13) and conditional *Sox9*-knockout epiphyses (GSM4930083, *Sox9* mutant-P13). The original study isolated articular and growth plate chondrocytes from dissected tibial and femoral epiphyses of control and *Sox9*-mutant mice. Loss of *Sox9* induces OA-like degeneration, whereas *Sox9* sufficiency protects articular cartilage from OA progression [52,53,54]. Accordingly, comparing these *Sox9*-null and WT datasets provides a relevant framework for evaluating *Ccn3*-positive cell states in healthy versus OA-prone cartilage.

Raw count matrices were processed in Seurat R package v.5.3.0. Cells were retained if they expressed 200–6000 genes and contained <10% mitochondrial transcripts. Data were log-normalized (NormalizeData) and scaled (ScaleData); 2000 highly variable genes were identified (FindVariableFeatures) and subjected to principal-component analysis (RunPCA). The first 30 principal components were used to generate uniform manifold approximation and projection (UMAP) embeddings (RunUMAP) for dimensional reduction.

Clusters were manually annotated based on canonical markers reported in the original study [52] (Figure S2). Additional markers from previously published literature were incorporated when necessary to aid in cluster identification. Eight clusters were identified: Chondrocytes, Osteoblasts (OBs), Immune Cells, Endothelial Cells, Osteoblast Progenitors (OBPs), Synovial Mesenchymal Stem Cells (SMSCs), Mesenchymal Stem Cells (MSCs), and Red Blood Cells (RBCs). Notably, *Ccn3* expressions were detected in three clusters: OBPs, SMSCs, and MSCs.

### 4.5. RNA Extraction from Articular Cartilage

The femoral heads of the knee joints were isolated for RNA extraction. Total RNA was purified using Isogen reagent (Nippon Gene Co., Tokyo, Japan), following the manufacturer’s protocol [8].

### 4.6. Cell Culture

Rat chondrosarcoma (RCS) cells were maintained in Dulbecco’s modified Eagle medium (DMEM) supplemented with 10% fetal bovine serum (FBS) at 37 °C in a humidified incubator with 5% CO_2_ [16]. Once the cells reached confluence, they were transfected with either *Ccn3*-specific siRNA (siRNA#SASI_Rn01_06106701; Sigma-Aldrich, St. Louis, MO, USA) or a non-targeting control siRNA (cat#SIC002; Sigma-Aldrich) by electroporation (Nucleofector, Basel, Switzerland), according to the manufacturer’s instructions. After 48 h, cells were treated with interleukin-1 beta (IL-1β, 5 ng/mL, Bio legend, San Diego, CA, USA). An additional 24 h later, total RNA and protein were collected for reverse transcription-quantitative PCR (RT-qPCR) and Western blot analyses. RNA was isolated using the RNeasy Mini Kit (Qiagen, Hilden, Germany), in accordance with the manufacturer’s guidelines.

### 4.7. Western Blot Analysis

Cultured cells were washed with PBS and lysed directly in a sodium dodecyl sulfate (SDS) sample buffer containing 0.125 M Tris-HCl (pH 6.8), 4% SDS, 20% glycerol, 2% β-mercaptoethanol, 0.002% (*w*/*v*) bromophenol blue. The lysates were boiled at 95 °C for 5 min, and equal amounts of protein were subjected to SDS-polyacrylamide gel electrophoresis, followed by transfer onto PVDF membranes (Millipore, Burlington, MA, USA). The membranes were blocked and incubated with primary antibodies against Ccn3 (provided by Dr. Takako Sasaki, Oita University) and β-actin (Sigma, St. Louis, MO, USA), followed by horseradish peroxidase-conjugated secondary antibodies.

### 4.8. Reverse Transcription and Quantitative Real-Time PCR

Total RNA was reverse transcribed into cDNA using the PrimeScript™ RT Reagent Kit (Takara Bio, Shiga, Japan) following the manufacturer’s protocol. Quantitative real-time PCR was subsequently carried out using Luna Universal qPCR Master Mix (New England Biolabs, Ipswich, MA, USA) on a StepOnePlus™ Real-Time PCR System (Applied Biosystems, Waltham, MA, USA). Reactions were run in triplicate. Gene expression levels were normalized to either *Gapdh* or *Actb*, used as internal controls to ensure equal cDNA input. Primer sequences and thermal cycling conditions were described previously [16].

### 4.9. Statistical Analysis

Data are expressed as individual values with the median and interquartile range (IQR) for ordinal variables and as mean ± standard deviation (SD) for continuous variables. Differences among multiple groups for continuous variables were analyzed by one-way ANOVA followed by Dunnett’s post hoc test using SPSS version 25, with statistical significance set at *p* < 0.05. For OARSI scores, which are ordinal, group differences were analyzed using the non-parametric Kruskal–Wallis test followed by Dunn’s multiple comparisons. Graphical representations were created with GraphPad Prism version 9.

## 5. Conclusions

Our in vivo and in vitro findings consistently demonstrate that *Ccn3* knockout or knockdown attenuates OA-associated cartilage degeneration by downregulating inflammatory and catabolic mediators, including *Mmp9*, *Mmp13*, *Adamts5*, and the hypertrophic marker *Col10a1*. Notably, *Ccn3* deficiency also increases the Ki67^+^ chondrocyte population during OA, suggesting enhanced proliferative potential in the absence of *Ccn3*. These results indicate that *Ccn3* plays a crucial role in OA pathogenesis by promoting matrix degradation, inducing hypertrophic changes, and restricting the proliferation of progenitor cells. Our findings highlight the induction of *Ccn3* in progenitor or stem-like cells within articular cartilage as a key event in OA progression, positioning it as a valuable therapeutic target for OA treatment.

## Data Availability

The datasets generated and analyzed during the current study are available from the corresponding author upon reasonable request.

## Supplementary Materials

The supporting information can be downloaded at: https://www.mdpi.com/article/10.3390/ijms26199630/s1.
