# Supplementary material for: Critical Requirement of Senescence-Associated CCN3 Expression in CD44-Positive Stem Cells for Osteoarthritis Progression
Source: Int J Mol Sci. 2025 Oct 2;26(19):9630. doi: 10.3390/ijms26199630 (PMC12524674; doi:10.3390/ijms26199630)
Supplement: Supplementary file 1 [file ijms-26-09630-s001.zip › ijms-3868687-supplementary.pdf]

Supplementary Figure S1. **PCR confirmation of Ccn3 knockout in mice.**

Genomic DNA from wild-type (WT) and *Ccn3* knockout (KO) mice were analyzed using genotype-specific primers. WT alleles were detected using *Ccn3* ko genotyping 5′/3′ primers, and KO alleles were detected using *Ccn3* ko neo genotyping 5′/3′ primers. The PCR results confirmed successful knockout of Ccn3 in KO mouse #53 and the presence of WT alleles in WT mice (#50, #51).

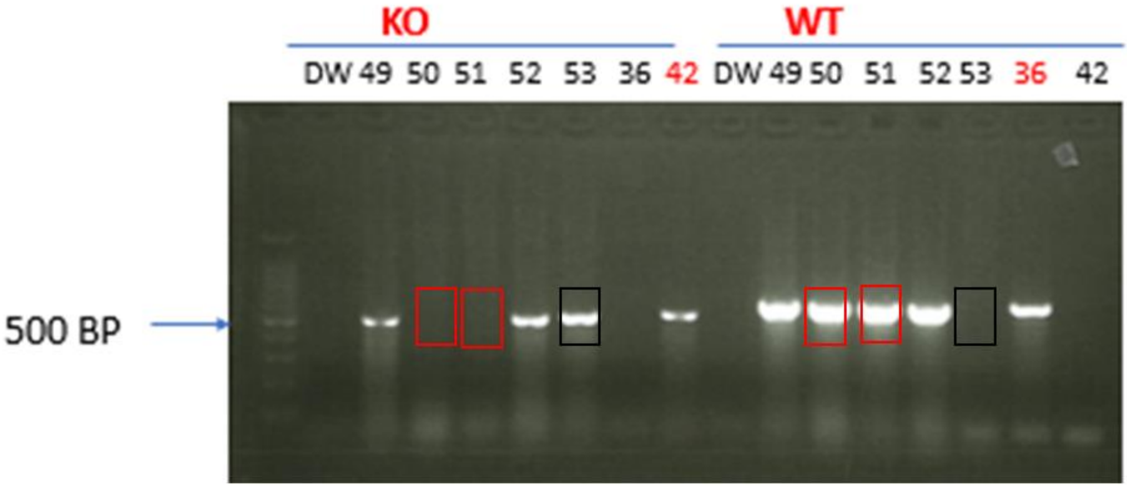

Supplementary Figure S2. Violin Plot of Key Cell Marker Expression Across Identified Cell Clusters.

This violin plot illustrates the expression patterns of key cell-type markers across 8 distinct cell clusters identified through single-cell RNA sequencing analysis. Each violin represents the distribution of gene expression within a cluster, with the width of the plot indicating the density of cells expressing that gene at different expression levels. The color of the plot reflects the genotype, with red indicating Sox9 mutant and blue indicating wild type. Marker genes associated with chondrocytes, progenitor cells, and other stromal or immune populations are included.

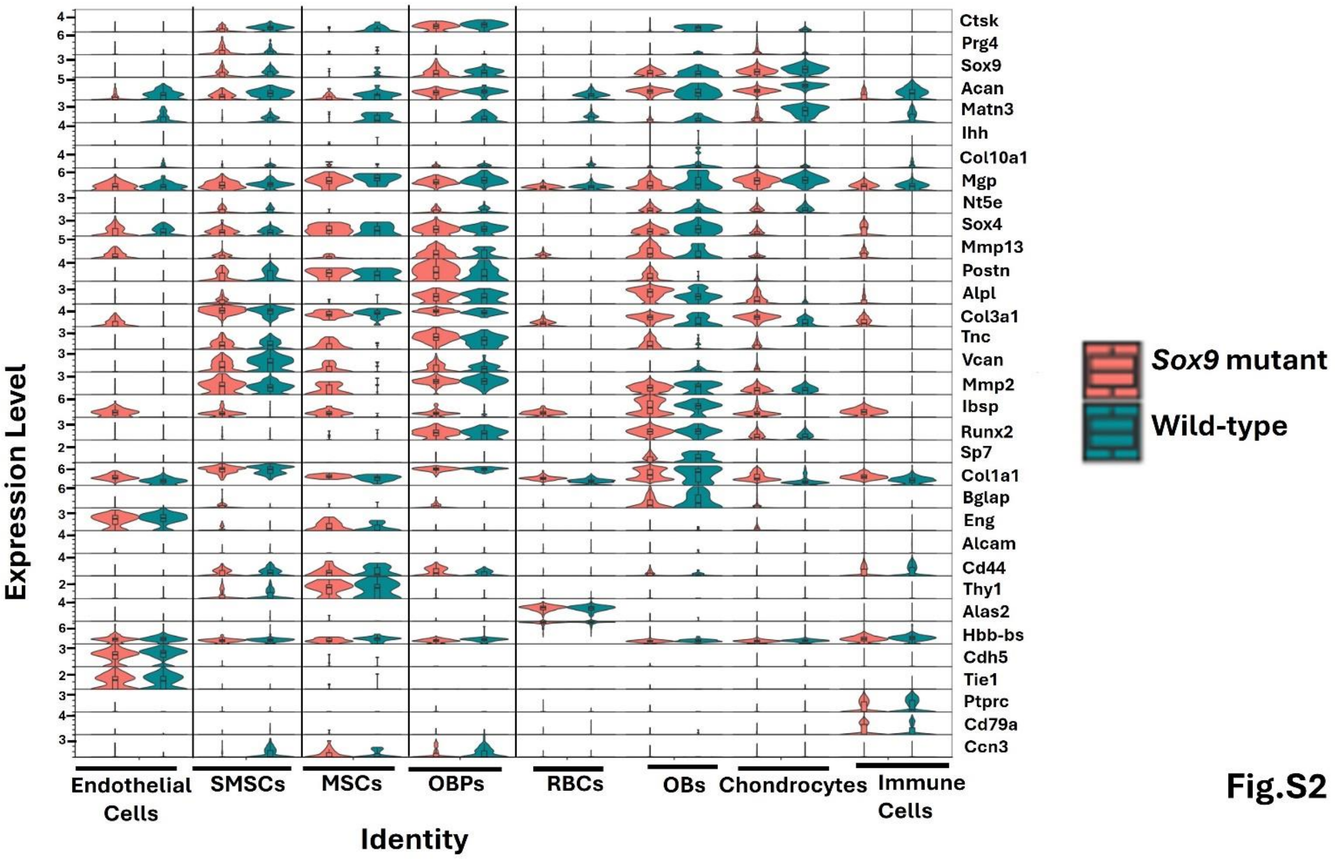

Fig.S2

Sup. Table S1. CT values for *Col10a1*

GROUP	MICE	CT	CT MEAN
WTDMM	132	26.2749	26.20962
	132	26.20042	
	132	26.15353	
	137	25.2917	25.30641
	137	25.27624	
	137	25.3513	
	342	26.42775	27.01977
	342	27.63415	
	342	26.99739	
	348	25.26467	24.96896
	348	24.68363	
	348	24.95859	
WT-SHAM	139	28.97865	28.52048
	139	28.20162	
	139	28.38119	
	141	29.55994	29.36289
	141	29.3378	
	141	29.19091	
	344	30.40976	30.61329
	344	31.76493	
	344	29.66519	
KODMM	129	29.30252	28.72127
	129	28.52167	
	129	28.33964	
	135	28.37507	28.61764
	135	28.52409	
	135	28.95375	
	133	29.60705	29.49937
	133	29.73773	
	133	29.15334	
KOSHAM	136	32.68211	30.43502
	136	28.6914	
	136	29.93154	
	130	24.3148	24.45826
	130	24.48515	
	130	24.57483	

Sup. Table S2. CT values for *Gapdh*

GROUP	MICE	CT VALUE	CT MEAN
WTDMM	132	26.67657089	27.45693
	132	27.75765419	
	132	27.93655396	
	137	30.45824051	30.52443
	137	29.76557732	
	137	31.34948158	
	342	30.18518066	30.23186
	342	30.52092552	
	342	29.98947906	
	348	28.9224472	28.83194
	348	28.80484581	
	348	28.76852798	
WT-SHAM	139	29.67116737	29.89726
	139	30.23273659	
	139	29.78788757	
	141	30.51954842	29.96264
	141	29.98530388	
	141	29.38306236	
	344	24.35004044	24.06728
	344	23.97195244	
	344	23.87985229	
KODMM	129	29.49211502	27.84516
	129	29.68754005	
	129	24.35581398	
	135	28.33988571	28.36379
	135	28.44303894	
	135	28.30845261	
	133	26.46092987	26.47704
	133	26.23982811	
	133	26.73034859	
KOSHAM	136	28.62666893	28.77761
	136	29.2730484	
	136	28.43312073	
	130	27.70931816	27.5765
	130	27.17116928	
	130	27.84902382	

Sup. Table S3. CT values for *Mmp9*

GROUP	MICE	CT	CT MEAN
WTDMM	132	28.49619	28.45432
	132	28.10817	
	132	28.7586	
	137	29.60694	29.38539
	137	29.30178	
	137	29.24745	
	342	33.4554	33.91761
	342	34.73509	
	342	33.56233	
	348	33.55089	32.97678
	348	33.41261	
	348	31.96684	
WT-SHAM	139	33.82842	35.95193
	139	34.31464	
	139	39.71273	
	141	Undetermined	35.5743
	141	36.29422	
	141	34.85439	
	344	33.68205	33.39536
	344	33.91486	
	344	32.58918	
KODMM	129	31.86959	31.65288
	129	31.65261	
	129	31.43642	
	135	31.42119	32.02563
	135	32.15351	
	135	32.50218	
	133	32.78767	34.10123
	133	35.81129	
	133	33.70472	
KOSHAM	136	32.30368	31.57437
	136	31.27272	
	136	31.14672	
	130	31.54912	31.27029
	130	31.03167	
	130	31.23009	

Sup. Table S4. CT values for *Ccn3*

GROUP	MICE	C_T	C_T Mean
WTDMM	132	32.35516357	32.60179
	132	33.3388176	
	132	32.11139297	
	137	34.70576859	33.10603
	137	32.56868362	
	137	32.04363632	
	342	Undetermined	32.25354
	342	32.1906929	
	342	32.31638336	
	348	35.63903809	35.19469
	348	35.70516586	
	348	34.23986435	
WT-SHAM	139	33.98271942	34.31273
	139	34.40100861	
	139	34.55445099	
	141	Undetermined	
	141	Undetermined	
	141	Undetermined	
	344	33.90377045	35.17997
	344	Undetermined	
	344	36.45617294	
KODMM	129	35.39753723	
	129	Undetermined	
	129	Undetermined	
	135	Undetermined	
	135	Undetermined	
	135	Undetermined	
	133	Undetermined	
	133	Undetermined	
	133	Undetermined	
KOSHAM	136	35.22197342	34.9889
	136	34.75582123	
	136	Undetermined	
	130	35.27405167	36.15564
	130	Undetermined	
	130	37.03722	

Sup. Table S5. CT values for P21

GROUP	MICE	CT	CT MEAN
WTDMM	132	29.57602	29.2368
	132	29.36345	
	132	28.77094	
	137	28.9285	28.9009
	137	28.91958	
	137	28.85462	
	342	30.14866	30.01776
	342	30.30603	
	342	29.59859	
	348	34.83835	33.4885
	348	32.13865	
	348	Undetermined	
WT-SHAM	139	33.83778	33.06597
	139	32.93007	
	139	32.43005	
	141	32.98314	32.59242
	141	31.39563	
	141	33.3985	
	344	33.61661	33.64999
	344	34.41474	
	344	32.91862	
KODMM	129	31.96574	31.35052
	129	30.91519	
	129	31.17062	
	135	30.79093	31.37233
	135	32.58915	
	135	30.7369	
	133	30.69776	30.68899
	133	30.46277	
	133	30.90643	
KOSHAM	136	34.33594	34.52804
	136	36.55699	
	136	32.6912	
	130	30.32746	30.81254
	130	31.15056	
	130	30.9596	

Supplementary Figure S3. Quantification of CCN3/CD44 Colocalization in WT-DMM

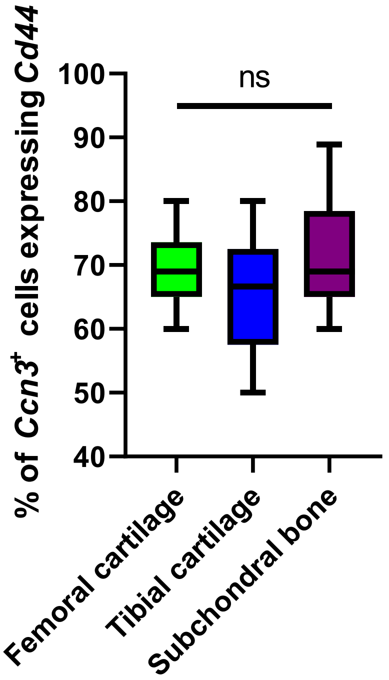

Cells were manually counted from immunofluorescence images using cellSens software (Olympus, Japan). C*cn3*-positive cells were identified first, and among these, cells co-expressing *Cd44* were recorded. The percentage of *Ccn3*+ cells also expressing *Cd44* was calculated by dividing the number of *Ccn3*/*Cd44* double-positive cells by the total number of $Ccn3^{+}$ cells in each field. Data are shown as box-and-whisker plots. Statistical analysis was performed using the Kruskal–Wallis test, which revealed no significant differences among regions ($p > 0.05$).
